# Clusterin/apolipoprotein J, its isoforms and Alzheimer's disease

**DOI:** 10.3389/fnagi.2023.1167886

**Published:** 2023-04-13

**Authors:** Giedre Milinkeviciute, Kim N. Green

**Affiliations:** ^1^Institute for Memory Impairment and Neurological Disorders, University of California, Irvine, Irvine, CA, United States; ^2^Department of Neurobiology and Behavior, School of Biological Sciences, University of California, Irvine, Irvine, CA, United States

**Keywords:** clusterin, apolipoprotein J, Alzheimer's disease, isoforms, mouse models

## Introduction

Late-Onset Alzheimer's Disease (LOAD) is the most common form of Alzheimer's disease, accounting for ~95% of all AD cases (Reitz and Mayeux, 2014). It is believed to be caused by a complex interplay of genetic and environmental factors. The Apolipoprotein E (ApoE) ε4 allele is the best-known genetic risk factor for LOAD (Jiang et al., 2013), but various genome-wide association studies (GWAS) have identified many other low-penetrance alleles that also influence the risk of LOAD. One such genetic factor is the clusterin (*CLU*) gene, also known as apolipoprotein J (ApoJ). This gene has been found to be one of the most important genetic factors associated with an increased risk of LOAD in multiple GWAS studies (Harold et al., 2009; Lambert et al., 2009; Seshadri et al., 2010; Wightman et al., 2021).

**Figure 1:**
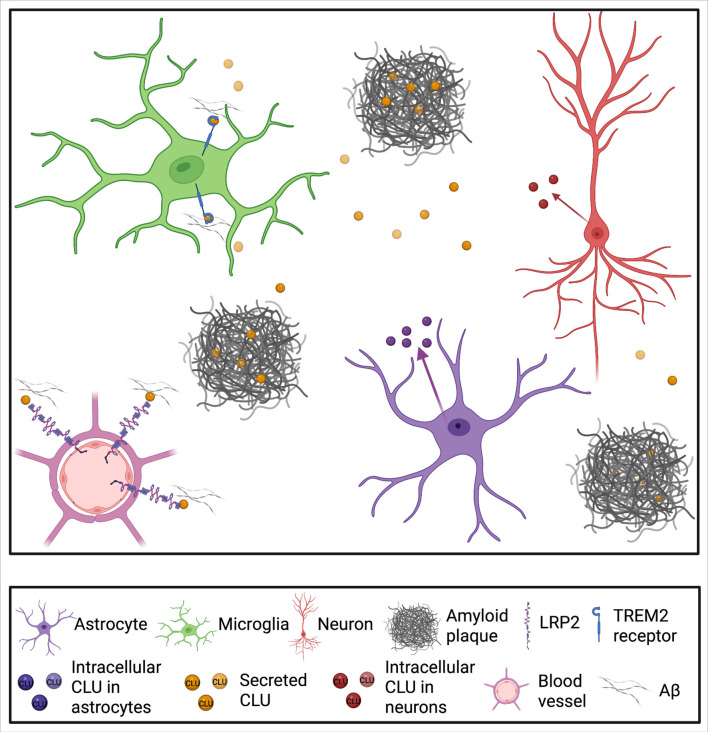
Clusterin (CLU) in the brain. *CLU* expression in the brain is found in astrocytes and neurons. CLU has multiple isoforms, including secreted and non-secreted. Secreted CLU is known to bind Aβ plaques as well as to participate in Aβ uptake by microglia *via* the TREM2 receptor and Aβ clearance *via* the brain vasculature. The figure was created with BioRender.com.

The CLU protein is found body wide, including peripheral organs (Ahuja et al., 1996; Guo et al., 2016; Park et al., 2020), the brain (Pasinetti et al., 1994; Thambisetty et al., 2013; Moon et al., 2021), and in bodily fluids (Trougakos and Gonos, 2002) such as plasma (De Silva et al., 1990b; Martinez-Bujidos et al., 2015; Hsu et al., 2017; Liu et al., 2021), urine (Solichova et al., 2007), cerebrospinal fluid (Nilselid et al., 2006), seminal fluid (Atlas-White et al., 2000; Saewu et al., 2017), and tears (Yu et al., 2018). The functions of CLU in peripheral tissues have been well studied and include the clearance of misfolded proteins (Humphreys et al., 1999), lipid transport (Calero et al., 1999), inhibition of the complement system (Jenne and Tschopp, 1989), and the regulation of oxidative stress and cell death (Foster et al., 2019). In the brain, CLU expression is found in astrocytes (Pasinetti et al., 1994; Morgan et al., 1995; Demattos et al., 2001; Charnay et al., 2008; John Lin et al., 2017; Chen et al., 2021) and in cortical and hippocampal neurons (Figure 1; O'bryan et al., 1993; Pasinetti et al., 1994; Herring et al., 2019). The specific functions of CLU in the brain, however, are not as well-understood. Studies have shown that CLU expression is upregulated in degenerative conditions, such as AD (Calero et al., 2005; Nuutinen et al., 2009), due to cellular and oxidative stress or dysregulation of specific signaling pathways (Wong et al., 1994; Gutacker et al., 1999; Schepeler et al., 2007; Trougakos and Gonos, 2009). However, the literature provides conflicting results as to whether CLU expression improves or exacerbates cellular stress (Schreiber et al., 1993; Han et al., 2001; Imhof et al., 2006; Kim et al., 2012; Trindade et al., 2016; Troakes et al., 2017).

In AD, levels of CLU are increased in the brain (Mcgeer et al., 1992; Lidstrom et al., 1998) and in cerebrospinal fluid (CSF) (May et al., 1990; Bertrand et al., 1995; Miners et al., 2017). CLU has been found to bind to amyloid-beta (Aβ) and play a role in Aβ deposition as well as its clearance (Wilson and Easterbrook-Smith, 1992; Narayan et al., 2011). CLU has also been found in Aβ plaques, vessels of cerebral amyloid angiopathy (CAA; Figure 1) (Mcgeer et al., 1992; Craggs et al., 2016; Camacho et al., 2019), associated with neurofibrillary

tangles (Mcgeer et al., 1992), and to interact with modified Tau species in human AD brain tissue (Zhou et al., 2014). However, CLU may also be involved in non-Aβ pathways that could alter susceptibility to AD (Braskie et al., 2011; Erk et al., 2011; Thambisetty et al., 2013). Importantly, different single nucleotide polymorphisms (SNPs) in the *CLU* gene may exert their effects in combination with other genetic risk factors, such as *APOE4* (Roussotte et al., 2014; Jackson et al., 2019), *TREM2* (Yeh et al., 2016) and *BIN1* (Zhou et al., 2014). Additionally, at least three different mRNA isoforms are produced from the CLU gene (Calero et al., 1999) and recent research suggests that different variants in the CLU gene may lead to alterations in the ratios of isoforms being produced, which in turn could affect the outcome of the disease and play a role in the development and progression of AD.

## CLU isoforms

Alternative splicing has been implicated in the susceptibility of Alzheimer's disease (AD) (Raj et al., 2018) and many genes associated with AD undergo alternative splicing (Rockenstein et al., 1995; Zhou et al., 2014; Koch, 2018), *CLU* being one of them (Szymanski et al., 2011; Foster et al., 2019; Herring et al., 2019; Han et al., 2020). *CLU* consists of 11 exons (two exons are untranslated) that give rise to secreted and cytosolic isoforms through alternative splicing. Research challenges exist due to the limited characterization of *CLU* mRNA transcript variants present in different sexes, at different developmental timepoints, cell types, and in different brain areas in a healthy brain (De Silva et al., 1990a; Herring et al., 2019).

CLU protein exists in multiple forms, including secreted (sCLU) and non-secreted/intracellular isoforms which are targeted to at least three different compartments within the cell (cytosol, ER/mitochondria, nucleus; Herring et al., 2019). Unless specified, here we will call all non-secreted isoforms intracellular CLU (iCLU). The different forms of CLU may have distinct functions, depending on where and when they are found, as well as how long they are produced. The sCLU is produced from Exon 2 (Rizzi and Bettuzzi, 2010; Ling et al., 2012) and contains an endoplasmic reticulum (ER) signaling peptide. Similar to the small heat-shock proteins, sCLU can function as a chaperone and bind to partially unfolded proteins preventing their aggregation (Humphreys et al., 1999; Wojtas et al., 2020). (Yeh et al., 2016; Wojtas et al., 2017; Yuste-Checa et al., 2021). iCLU forms have been suggested to play a role in apoptosis (Yang et al., 2000; Debure et al., 2003; Zhang et al., 2005), DNA repair (Yang et al., 2000), transcription (Santilli et al., 2003) and microtubule organization (Kang et al., 2005). One of the iCLU is produced from Exon 3 and lacks Exon 2 (Leskov et al., 2003; Kim et al., 2012; Prochnow et al., 2013), while another iCLU isoform lacks exon 5 (Kimura and Yamamoto, 1996; Kimura et al., 1997; Leskov et al., 2003). sCLU has been suggested to promote cell survival (Trougakos and Gonos, 2009), while iCLU is associated with decreased cell growth and apoptosis (Yang et al., 2000; Scaltriti et al., 2004; Kim and Choi, 2011). The iCLU that lacks exon 2 has been termed a nuclear CLU (nCLU) and described as a cell death protein that can be found in the cytoplasm and nuclei of cells (Kimura and Yamamoto, 1996; Yang et al., 2000; Leskov et al., 2003; Prochnow et al., 2013). However, this isoform is present at very low levels (Yang et al., 2000; Prochnow et al., 2013) or, as some state, is not produced at all (Andersen et al., 2007). It is worth noting that many studies on the role of CLU isoforms have been conducted *in vitro*, in cancer cells, and not in the healthy brain (Leskov et al., 2003; Rodriguez-Pineiro et al., 2006; Moretti et al., 2007; Rizzi and Bettuzzi, 2010).

In the most comprehensive study of *CLU* isoforms so far, *CLU* mRNA transcripts have been characterized in the rodent brain, primary cultures of rodent and human neurons and astrocytes, and in rodent and human brain-derived cell lines (Herring et al., 2019). In the cortex, iCLU could be found in the nuclear, organelle, and cytosolic compartments of neurons, but only low levels were detected in astrocytes. Six different CLU immunoreactive bands (5 in cytosolic and 1 in nuclear fraction) were identified in primary cultures of rodent cortical neurons. Five immunoreactive bands (4 in cytosolic and 1 in nuclear fraction) were detected in astrocytes. It was concluded that two isoforms were exclusive to neurons. Three different Exon 1 variants were discovered—Exon 1A, Exon 1B, and Exon 1C. Exon 1A and Exon 1C are expressed in astrocytes and neurons, respectively, while Exon 1B mRNA transcript was detected in both cell types and, thus, two neuron-specific CLU isoforms likely originate from a neuron-specific Exon 1C variant. A novel mitochondrial CLU (mitoCLU) was identified in female adult cortical tissue, which is translated from a non-canonical start site CUG (Leucine) in Exon 3. mitoCLU is also found in human cells but is generated from an AUG and a CUG start sites located in Exon 3 (Herring et al., 2019). In light of these findings, there may be a need to re-evaluate some of the historical results.

## CLU isoforms in AD

Multiple *CLU* SNPs, both intronic and exonic, have been associated with LOAD (Harold et al., 2009; Lambert et al., 2009; Moon et al., 2021). Interestingly, while some confer protection against AD, others have been linked to an increased AD risk [reviewed in Woody and Zhao (2016)]. Both sCLU and nCLU are upregulated in response to stress (Nizard et al., 2007) and inflammation, and CLU and CLU mRNA is altered in neurodegenerative disorders (Grewal et al., 1999; Sasaki et al., 2002; Ingram et al., 2014; Labadorf et al., 2015; Das Gupta et al., 2019; Yuste-Checa et al., 2022), including AD (Calero et al., 2005; Zhou et al., 2014; Bettens et al., 2015; Foster et al., 2019; Jackson et al., 2019). However, while sCLU is thought to be protective, iCLU isoforms were linked to cytotoxicity (Nizard et al., 2007; Prochnow et al., 2013; Yeh et al., 2016; Wojtas et al., 2017; Yuste-Checa et al., 2021). Therefore, CLU variants and other factors that modify the ratio between different isoforms could also alter the risk for LOAD and associated brain pathology through the multiple functions attributed to CLU.

The most well-known SNP is rs11136000, which is located in intron 3 and is carried by about 36% of the Caucasian population (Bertram et al., 2007; Braskie et al., 2011). The major allele, rs1113600^C^, is associated with reduced expression of *CLU* and an increased risk of AD (Ling et al., 2012; Roussotte et al., 2014; Tan et al., 2016). This allele has also been linked to faster cognitive decline (Thambisetty et al., 2013) and poorer memory scores (Pedraza et al., 2014). Additionally, research has shown that the C allele can change brain structure and network activity in young adults, suggesting that brain circuitry in early life may contribute to cognitive effects later in life (Braskie et al., 2011; Lancaster et al., 2015). In contrast, the minor allele, rs11136000^T^, is associated with increased *CLU* expression and a reduced risk of AD (Ling et al., 2012; Roussotte et al., 2014; Tan et al., 2016). Studies have also found that this allele is associated with increased nCLU expression, but not sCLU. The ratio of expressed isoforms may change depending on the CLU genotype, with both nCLU and sCLU levels increasing with AD (Ling et al., 2012).

Other SNPs have also been linked to changes in CLU protein localization, for example, rare SNPs located in exons 5 and 6 have been identified and linked to alterations in CLU isoform production in AD, such as a reduction in sCLU (Bettens et al., 2012, 2015; Han et al., 2020). Studies with Tg4510 mice, which overexpress the human mutant P301L Tau (Ramsden et al., 2005), have also shown changes in CLU expression. In these mice, sCLU was upregulated in the hippocampus at 5.5 months, but a truncated version of iCLU was increased in the hippocampus as early as 2 months (pre-tangle time point). This truncated version of iCLU was found to interact directly with Tau protein, but outside of the microtubule binding region. Interestingly, the Tg2576 mouse model of amyloidosis did not show any age-related changes in CLU isoform expression (Zhou et al., 2014) suggesting that in this study Tau rather than amyloid was driving changes in CLU isoform expression.

## CLU function in AD

The availability of CLU mouse models is currently limited to two models from the Jackson Laboratory. The first model is a CLU^−/−^ model (JAX:005642) that was developed over 20 years ago (Mclaughlin et al., 2000). The second model contains a 2kb region of human DNA sequence that spans from intron 7 to exon 9, including a human LOAD *CLU* risk SNP rs2279590 (JAX:037496), that we have produced as part of the MODEL-AD consortium that is currently undergoing phenotyping. Recently, it was discovered that the existing CLU^−/−^ model is not a complete KO as the mitoCLU isoform is still present (Herring et al., 2019). In addition, a mouse with the deletion of exon 3 was created in 2021, but, except for the effect on the auditory function, has not been characterized yet or validated (Zhao et al., 2021). Given the association between *CLU* risk alleles and altered CLU isoform production (Foster et al., 2019; Han et al., 2020), there is a significant urgency to validate CLU functions identified with the currently available CLU^−/−^ model in the Jackson Laboratory, and possibly to create the new CLU^−/−^.

The function of CLU in AD has been studied using mouse models, including the CLU^−/−^ model and its crosses with other known AD models. Studies have shown that loss of CLU in PDAPP transgenic mice leads to a reduction in dense core plaques and neuritic dystrophy (Demattos et al., 2002), while crossing of the same CLU^−/−^ with APP/PS1 mice increased incidence of cerebral amyloid angiopathy while also reducing dense core plaques (Wojtas et al., 2017). Notably, CLU^−/−^ mice show impaired presynaptic function, and reduced spine density (Chen et al., 2021). CLU^−/−^ crossed with the 5xFAD mouse model of amyloidosis led to decreased levels of soluble Aβ oligomers and amyloid plaques and an increase in synaptic proteins as well as improved scores of behavioral tests. However, these results were only seen in younger mice, suggesting CLU's role in the early stages of AD (Oh et al., 2019). However, due to incomplete KO of all CLU isoforms in the CLU^−/−^ mice used in all of these studies, these findings need to be re-explored. Additionally, some data on CLU function is available through CLU overexpression studies, where overexpression in astrocytes of 5xFAD mice reduced amyloid pathology, neuronal toxicity, and rescued synaptic deficits (Chen et al., 2021). A recent publication also describes the effects of removing Exon 2 from *CLU* and shows downregulation of extracellular matrix pathways in cultured neurons (Foster et al., 2022). However, no AD-related *CLU* mutations have been identified in Exon 2, which harbors the ER-targeting sequence (Moon et al., 2021; Foster et al., 2022).

There are two ways that CLU has been suggested to be involved in the Aβ clearance – transvascular and microglial. Transvascular pathway encompasses Aβ clearance across the blood-brain barrier (BBB) as a free peptide and/or bound to APOE or CLU. While APOE2 and APOE3 bound Aβ is removed via endothelial low-density lipoprotein receptor-related protein 1 (LRP1), CLU facilitates Aβ clearance via endothelial low-density lipoprotein receptor-related protein 2 (LRP2) (Bell et al., 2007; Zlokovic, 2011; Zhao et al., 2015b). APOE4 cannot bind LRP1 and, thus, together with aging, leads to enhanced risk of cerebral amyloid angiopathy (CAA) (Zhao et al., 2015a,b). In 12 month *APP/PS1;CLU*^−/−^ mice, significant increase in dense core Aβ plaques was observed in leptomeningeal vessels and penetrating arterioles. Accordingly, plaques were reduced in cortical and hippocampal regions indicating a shift to Aβ accumulation in the perivascular drainage pathways leading to increased CAA in the absence of CLU. This study also showed an increase in Aβ40:42 ratio due to a longer Aβ40 clearance time in mice lacking *CLU* (Wojtas et al., 2017). Similarly, in 12 month *PDAPP;CLU*^−/−^ mice, a reduction in Aβ plaques was reported, but no changes in the total Aβ levels in the cortex or hippocampus or effect on CAA. Interestingly, authors did observe an increase in soluble Aβ in the brain (Demattos et al., 2004). It was suggested that the differences in effect on CAA may be due to the younger age that the mice develop CAA at, which is 6 and 12 months for the *APP/PS1, PDAPP* mice, respectively (Nelson et al., 2017). Overall, CLU plays a role in the Aβ clearance via the brain vasculature, thus, SNPs in *CLU* leading to changes in the levels of secreted CLU, may affect the severity of CAA observed in AD. Given that Aβ40 is the predominant Aβ species which accumulates in the vessel walls and presents as CAA (Yamada, 2015; Robert et al., 2017), lack of CLU may be responsible for the altered clearance of Aβ40 specifically and, in turn, the enhanced risk of CAA.

CLU and APOE have been identified as ligands of the triggering receptor expressed on myeloid cells 2 (TREM2) (Yeh et al., 2016). TREM2 is a receptor that is selectively expressed on microglia in the brain (Wang et al., 2015; Ulland et al., 2017; Nugent et al., 2020) and macrophages in the periphery (Chung et al., 2002), and is known to play a role in inflammatory signaling (Kobayashi et al., 2016), microglial metabolism (Ulland et al., 2017), phagocytosis (Poliani et al., 2015; Wang et al., 2015), activation (Jay et al., 2015; Wang et al., 2015), survival (Wang et al., 2015; Ulland et al., 2017; Zheng et al., 2017), and proliferation (Poliani et al., 2015). TREM2 was identified as a lipid receptor and was shown to control cholesterol and phospholipid metabolism in the brain (Wang et al., 2015; Andreone et al., 2020; Nugent et al., 2020; Li et al., 2022). Lipidated CLU can bind to Aβ, and CLU-Aβ complexes can then be taken up by microglia through binding to TREM2 (Yeh et al., 2016). This suggests that CLU may be facilitating microglial Aβ uptake through TREM2 (Figure 1), and a lack of sCLU due to changes in CLU isoform expression may affect Aβ uptake and clearance. In addition, given the role that lipid metabolism plays in AD (Zhu et al., 2019; Kao et al., 2020; Paasila et al., 2021; Turri et al., 2022), TREM2 function as a lipid metabolism regulator (Wang et al., 2015; Nugent et al., 2020; Li et al., 2022), and CLU binding lipids and cholesterol to influence their trafficking (Matukumalli et al., 2017; Foster et al., 2019), the interaction between TREM2 and CLU should be further explored as it could have a great impact on AD pathogenesis.

## Conclusions

In conclusion, the role of CLU in Alzheimer's disease is complex and not fully understood. Multiple CLU isoforms have been identified, but future research is needed to understand their transcription patterns in different cell types, brain regions, during development and aging, in healthy brain and disease as well as different sexes. Moreover, it is still unclear how each identified AD-linked *CLU* SNP alters the ratios of translated CLU isoforms. Studies utilizing a single CLU^−/−^ model and crossing it with other known mouse AD models have shown a range of CLU functions in the AD brain. However, these studies may need to be re-evaluated due to incomplete KO of all the CLU isoforms in this model. New and improved CLU models focusing on different CLU isoforms are needed to elucidate the specific role of CLU in healthy brain, the development and progression of AD.

## Author contributions

GM wrote the first draft of the manuscript. KNG edited the manuscript. Both authors have read and approved the final manuscript for publication.
